# Gold and silver nanoparticles for biomolecule immobilization and enzymatic catalysis

**DOI:** 10.1186/1556-276X-7-287

**Published:** 2012-06-01

**Authors:** Galina A Petkova, Кamil Záruba, Pavel Žvátora, Vladimír Král

**Affiliations:** 1 Department of Analytical Chemisty, Institute of Chemical Technology in Prague, Technicka 5, Dejvice, Prague 6,, 166 28, Czech Republic; 2 Zentiva (part of the Sanofi-Aventis Group), U Kabelovny 130, Dolni Mecholupy, Prague 10,, 102 37, Czech Republic

**Keywords:** Enzyme immobilization, Cofactor immobilization, Gold and silver nanoparticles, Spectroscopies, conformational change, Enantioselectivity

## Abstract

In this work, a simple method for alcohol synthesis with high enantiomeric purity was proposed. For this, colloidal gold and silver surface modifications with 3-mercaptopropanoic acid and cysteamine were used to generate carboxyl and amine functionalized gold and silver nanoparticles of 15 and 45 nm, respectively. Alcohol dehydrogenase from *Thermoanaerobium brockii* (TbADH) and its cofactor (NADPH) were physical and covalent (through direct adsorption and using cross-linker) immobilized on nanoparticles' surface. In contrast to the physical and covalent immobilizations that led to a loss of 90% of the initial enzyme activity and 98% immobilization, the use of a cross-linker in immobilization process promoted a loss to 30% of the initial enzyme activity and >92% immobilization. The yield of NADPH immobilization was about 80%. The best results in terms of activity were obtained with Ag-citr nanoparticle functionalized with carboxyl groups (Ag-COOH), Au-COOH(CTAB), and Au-citr functionalized with amine groups and stabilized with CTAB (Au-NH_2_(CTAB)) nanoparticles treated with 0.7% and 1.0% glutaraldehyde. Enzyme conformation upon immobilization was studied using fluorescence and circular dichroism spectroscopies. Shift in ellipticity at 222 nm with about 4 to 7 nm and significant decreasing in fluorescence emission for all bioconjugates were observed by binding of TbADH to silver/gold nanoparticles. Emission redshifting of 5 nm only for Ag-COOH-TbADH bioconjugate demonstrated change in the microenvironment of TbADH. Enzyme immobilization on glutaraldehyde-treated Au-NH_2_(CTAB) nanoparticles promotes an additional stabilization preserving about 50% of enzyme activity after 15 days storage. Nanoparticles attached-TbADH-NADPH systems were used for enantioselective (*ee* > 99%) synthesis of (*S*)-7-hydroxy-2-tetralol.

## Background

Immobilization process has been used for enhancing enzyme activity and stability in aqueous media [1] and more recently in non-aqueous media [2-4]. Enzyme immobilization has attracted interest in biotechnological processes due to its high operational stability and durability, easy separation of products, and low cost of industrial applications [5]. A wide variety of immobilization techniques can be used, including adsorption on solid supports [6,7], covalent attachment [8,9], or entrapment in silica/polymer matrices [2]. Immobilization techniques are usually very easy to apply, but the bonding of the biocatalyst to the surface of the matrix is relatively weak. This leads to the leaching of the enzyme with conformational changes [10,11]. Covalent attachment normally leads to the improved enzyme stability, often at the cost of the partial inactivation due to the conformational changes induced by the covalent bonding of the enzyme residues to the matrix [12]. However, the immobilization on solid surface induces structural changes which may affect the entire molecule. The physico-chemical properties of the support may induce different conformational state in immobilized enzyme [13]. Enzyme 3D structure is essential to its activity. Any structural alteration, namely unfolding of the protein, is responsible for the loss of activity [7]. Circular dichroism and intrinsic fluorescence are two of the most sensitive tools for studying protein conformation [14].

Selecting support matrix and designing the carrier are very important in enzyme immobilization. In recent years, nanostructured materials have found a broad application as a matrix for enzyme immobilization. Among the various nanostructures (silica, carbon nanotubes, metal nanoparticles, and others), silver and gold nanoparticles are very attractive to be used as host matrixes. They are spherically shaped with a diameter of several tens of nanometers in size, comparable to the large biological molecules such as enzymes, antibodies, and receptors. With the size of thousand times smaller than cells, these nanoparticles offer a variety of biomedical applications including cancer diagnosis [15], radiotherapy [16], drug and gene delivery [17], sensors and biosensors [18], and controlling and suppressing of bacterial growth [19]. Ag and Au nanoparticles with large surface area and good electronic properties may provide a stable surface for enzyme immobilization. They can act as conduction centers to facilitate transfer of the electrons. Immobilization of the redox enzymes together with colloidal gold/silver is thought to either help the protein to assume a favorable orientation or to make possible conducting channels between the prosthetic groups and the gold/silver surface [20]. Because the gold and silver surfaces permit absorption of protein molecules, silver and gold nanoparticles have been used as a matrix for enzyme immobilization where the activity of enzymes is retained [12,21]. Enzymatic immobilization on solid supports as Au and Ag nanoparticles are done using either as whole cells or isolated enzymes, which include lysozyme [22], glucose oxidase [23,24], aminopeptidase [21], as well as alcohol dehydrogenase [25].

Alcohol dehydrogenases have long been used in industry as catalysts for catabolic processes or for the production of chemical enantiomers. Enzymes show good capacity as a redox biocatalysts in a variety of stereoselective reductions [26] and *in vivo* regeneration of a cofactor NAD(P)H [27]. Alcohol dehydrogenase from *Thermoanaerobium brockii* (TbADH) is a NADP-linked dehydrogenase. The enzyme is a tetramer of four identical polypeptide chains, each composed of 352 amino acids and 38 kDa [28]. TbADH preferably oxidizes secondary alcohols [29,30]. The enzyme has been immobilized by physical and chemical methods, namely adsorption on silica [31,32]. To facilitate the reuse of expensive cofactor, efforts have been made in regenerative enzyme-cofactor system both immobilized on the same matrix [33-35]. Construction of such system is essential to choose appropriate support that retains the cofactor while allowing the substrate and product to pass through. Enzyme-cofactor-particle preparations are much easier to reuse and afford more flexible reactor design.

Here, we have prepared functionalized gold and silver nanoparticles for the immobilizations of TbADH and its cofactor NADPH. The aim was to study the effect of different preparations (physical, covalent, and cross-linking immobilizations) on enzyme activity and structure. Size of the nanoparticles and structure of the enzyme before and after their interaction were characterized by TEM, circular dichroism, and fluorescence spectroscopies. Cofactor binding, activity measurements, storage stability, and ability of the nanoparticle-immobilized TbADH-NADPH bioconjugates to synthesize chiral alcohols with high enantioselectivity using 7-hydroxy-2-tetralone as substrate were also studied.

## Methods

### **Reagents**

Alcohol dehydrogenase from *T. brockii* (EC 1.1.1.2), NADPH, silver nitrate (99%), and K[AuCl_4_] (98%) were purchased from Sigma-Aldrich, St. Louis, MO, USA. Sodium citrate (99%) was purchased from Penta, Chrudim, Czech Republic. 3-Mercapotpropanoic acid (99%), bovine serum albumin, and glutaraldehyde (25%) were purchased from Fluka, Sigma-Aldrich Corporation, St. Louis, MO, USA. 7-Hydroxy-2-tetralone was donated from Zentiva Group, Prague, Czech Republic. Ultrapure water (*R* = 10 MΩ) was purchased from Milli-Q system, Prague, Czech Republic. All other reagents and solvents were of analytical grade purchased from commercial suppliers and used without further purification.

### **Preparation of gold and silver nanoparticles**

#### ** *Preparation of silver nanoparticles* **

Silver nanoparticles were prepared by citrate-reduction of AgNO_3_[36]. Resulting nanoparticles were marked citrate-stabilized silver (Ag-citr) nanoparticles.

*Ag-citr nanoparticles functionalized with carboxyl groups:* 10 mL of Ag-citr solution was treated with 100-μL stock solution of 3-mercaptopropanoic acid (MPA) (7.13-μL MPA dissolved in 2-mL distilled water) to give modified silver nanoparticles, marked Ag-citr nanoparticle functionalized with carboxyl groups (Ag-COOH).

#### ** *Preparation of gold nanoparticles* **

Gold nanoparticles were prepared by citrate-reduction of K[AuCl_4_[37]. Resulting nanoparticles were marked citrate-stabilized gold (Au-citr) nanoparticles.

*Au-citr nanoparticles stabilized by cetyltrimethylammonium bromide (CTAB):* To a 15-mL Au-citr nanoparticles, 20-μL (0.1 M) CTAB were added. Resulting nanoparticles were marked Au-citr(CTAB).

*Au-citr nanoparticles functionalized with carboxyl groups:* To a 100-mL solution of Au-citr(CTAB), 6.3-μL MPA was added. The resulting solution was left to stand for 3 days in the dark at 26°C. Resulting nanoparticles were marked Au-citr nanoparticle functionalized with carboxyl groups (Au-COOH). Then carboxyl modified nanoparticles were treated with 1.5-mL (0.1 M) CTAB. Resulting nanoparticles were marked cetyltrimethylammonium bromide (CTAB)-stabilized Au-COOH (Au-COOH(CTAB)).

*Au-citr nanoparticles functionalized with amine groups and stabilized with CTAB:* 15 mL of Au-citr(CTAB) solution was mixed with 20-μL (5.8 mg mL^−1^) cysteamine and left to stand for 2 days in the dark. Then, thiol modified nanoparticles were treated with 1.5-mL (0.1 M) CTAB. Resulting nanoparticles were marked Au-citr functionalized with amine groups and stabilized with CTAB (Au-NH_2_(CTAB)).

*Glutaraldehyde-activated Au-NH*_*2*_*(CTAB) nanoparticles:* 6 mL of Au-NH_2_(CTAB) solution was centrifuged (10 min at 10,000 rpm), washed with 10-mM phosphate buffer, pH 7.0, and divided into aliquots of 1 mL each. The solutions were treated with 0.3%, 0.5%, 0.7%, 1.0%, 1.4%, and 1.8% of 25% (*v*/*v*) glutaraldehyde (GA) under continuous stirring for 17 h at 26°C [9]. Then, the solutions were centrifuged (10 min at 10,000 rpm) and washed with fresh phosphate buffer to remove any excess of GA molecules. Resulting nanoparticles were marked Au-NH_2_(CTAB) treated with glutaraldehyde (Au-(*n*%)glut) (*n* means the percent (*v*/*v*) of GA used in the treatment).

Finally, functionalized Ag and Au nanoparticles were washed with fresh 10-mM phosphate buffer, pH 7.0 by centrifugation/re-dispersion cycle twice (10 min at 10,000 rpm), and working solutions were stored in the dark at 26°C for the further reactions.

### **Preparation of bioconjugates**

#### ** *TbADH bioconjugates* **

Au-citr(CTAB) nanoparticles were used in the physical immobilization. For this, 3-mL Au-citr nanoparticles were well dispersed by ultrasonic before use and then 3-mg enzyme solution was added under continuous stirring (300 rpm) for 7 h at 26°C. An aliquot (100 μL) was periodically taken, and enzyme activity and protein loading were checked. Finally, Au-citr(CTAB)-TbADH bioconjugate was washed repeatedly with 10-mM phosphate buffer, pH 7.0, and the amount of immobilized enzyme was calculated from the difference in the protein amount before and after immobilization process.

The procedures for preparing Ag-COOH-TbADH, Au-COOH-TbADH, and Au-COOH(CTAB)-TbADH bioconjugates were the same as for Au-citr(CTAB)-TbADH. The procedure of preparing Au-(*n*%)glut-TbADH bioconjugates was similar to those of Au-citr(CTAB)-TbADH except that 2-mg TbADH solution was mixed with 1 mL of each Au-(*n*%)glut solution.

The amount of immobilized TbADH on the gold and silver nanoparticles was determined by substracting the amount of unbound protein from the protein originally added, using the Bradford assay method [38].

#### ** *NADPH bioconjugates* **

Two milligrams of (1.6 mM) NADPH were added to a 1-mL solution of functionalized Ag and Au nanoparticles, respectively, and were left under continuous stirring for 7 h at 26°C [34]. The unbound cofactor molecules were removed by centrifugation (10 min at 10,000 rpm) and washed with 10-mM phosphate buffer, pH 7.0. The amount of NADPH immobilized on nanoparticles was determined from the difference in absorbance at 340 nm before and after immobilization process.

### **Activity and storage stability measurements**

The activity of TbADH was determined by measuring the initial reaction rate of 7-hydroxy-2-tetralone following the decrease of NADPH concentration at 340 nm on Cary 400 UV–vis spectrometer (Varian Inc., Palo Alto, CA, USA). In 2 mL of 10-mM phosphate buffer (pH 7.0), 0.2-mM free or immobilized NADPH, 10-mM 7-hydroxy-2-tetralone, 1% (*v*/*v*) 2-propanol (for dissolving of substrate and cofactor regeneration), and 20 μL (about 40 μg) of the free or immobilized enzyme were mixed. Reaction mixture in the absence of enzyme was used as a blank. One TbADH unit was defined as the amount of the enzyme that reduced 1-μmol NADPH/min under experimental conditions.

Residual activity was obtained from the difference between activity in the suspension and the supernatant in relation to the initial activity of the process. Relative activity was calculated using the residual activity at each time point relative to that found initially on day 0.

For the storage stability measurements, 20 to 25 μg of the free and immobilized enzyme was incubated for 15 days in 10-mM phosphate buffer, pH 7.0 at 4°C. The residual activity was determined every 2 days as described above.

### **Characterization**

The average particle size and morphology of the nanoparticles were observed by TEM using JOEL microscope (JEM-1010, Santa Barbara, CA, USA) with accelerating voltage of 100 kV.

*Circular dichroism (CD)* spectra were recorded on Jasco 400 spectrophotometer (JASCO International Co., Ltd., Hachioji, Tokyo, Japan) at 25°C in the range of 300 to 200 nm. Quartz cuvette with 1-cm optical path length was used. Molar ellipticity (*θ*, deg cm^2^ dmol^−1^) was measured every 2 s with an integration time of 50 nm min^−1^. At least three full wavelength scans were collected and averaged for each sample. The spectra were recorded to characterize possible interaction between the nanoparticles and the enzyme. Secondary structure of TbADH, before (140-μg mL^−1^ enzyme) and after (1-mg mL^−1^ enzyme) immobilizations on nanoparticles, was determined by comparing the molar ellipticity at 222 nm (*θ*_222_). CD spectra for both the native enzyme and functionalized Ag and Au nanoparticles (1 mL) were recorded as a control.

*Intrinsic tryptophan fluorescence* spectra were recorded on Fluoro Max 2 fluorimeter (HORIBA Jobin Yvon Inc., Edison, NJ, USA) at 25°C. Fluorescence within native and immobilized enzyme was monitored at excitation at 285 nm and emission at 340 nm. Fluorescence spectra for both the native enzyme (140 μg mL^−1^) and functionalized Ag and Au nanoparticles (1 mL) were recorded as a control. Sample of 1 mg mL^−1^ of immobilized enzyme was used in order to characterize possible interaction between the nanoparticles and the enzyme.

### **Biotransformations with TbADH immobilized on Ag and au nanoparticles**

Biotransformation studies with nanoparticle-attached enzyme-cofactor systems were also provided. About 675 μg mL^−1^ of the free or immobilized TbADH was mixed with 0.5 mM of the free or immobilized NADPH and 10-mM 7-hydroxy-2-tetralone (dissolved in 2% (*v*/*v*) 2-propanol) under stirring for 48 h at 30°C. At determined time intervals, a sample (100 μL) was taken for RP-HPLC analyses. Finally, the reaction mixture was extracted with ethyl acetate, concentrated, and purified for chiral HPLC analyses.

RP- and chiral HPLC conditions for the separation of 7-hydroxy-2-tetralone and (*S*)-7-hydroxy-2-tetralol are described in Additional file 1.

## Results and discussion

### **Characterization of silver and gold nanoparticles**

UV–vis spectra and TEM images of citrate-stabilized Ag and Au nanoparticles, of 45 and 15 nm, respectively, are shown in (Additional file 2: Figure S1).

### **Immobilizations of TbADH and cofactor NADPH on Ag and Au nanoparticles**

#### ** *TbADH immobilization* **

**Figure 1:**
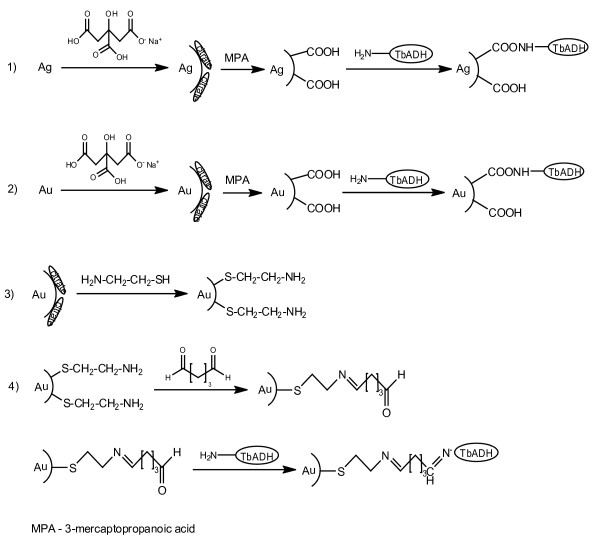
Surface modification of Ag-citr and Au-citr nanoparticles and immobilization of TbADH.

In this paper, Ag and Au nanoparticles functionalized with carboxyl and amine groups were employed as carriers for TbADH immobilization (Figure 1). Citrate reduced Ag and Au nanoparticles have a tendency to aggregate depending on the environment. To avoid such aggregations Ag-citr and Au-citr nanoparticles were protected with long aliphatic thiol with carboxyl and amine groups and then stabilized with CTAB. Also, to improve protection of the nanoparticles in the immobilization process 10-mM phosphate buffer, pH 7.0 was used.

The enzyme was immobilized on Ag and Au nanoparticles by physical and covalent (direct and through cross-linker) adsorption, which was ensured by reactive amine and carboxyl groups of the enzyme [38]. The process of evolution was followed by measuring the total protein in the supernatants compared to the blank control. The time course of immobilizations followed the similar way (data not shown). In all experiments, the immobilizations were almost completed in less than 3 h. However, reaction time of 7 h was chosen to ensure a maximum loading of the enzyme on the nanoparticles. Some of the characteristics of the free enzyme were compared to those of the immobilized enzyme under experimental conditions, which are summarized in Tables 1 and 2.

**Table 1:** Characterizations of TbADH and NADPH immobilized on functionalized Ag and Au nanoparticles

**Nanoparticles' system**	**Enzyme loading** **(mg/mL) (%)**	**Activity expressed (U/mL)**	**Specific activity (U/mg)**	**C**_**NADPH**_**, mM**
Free TbADH	-	0.5	12.5	-
Free NADPH	-	-	-	1.6
Ag-COOH^a^	2.9 (97)	0.23	5.8	1.28
Au-citr(CTAB)^b^	2.9 (98)	0.04	1.1	1.2
Au-COOH^b^	2.9 (98)	0.14	3.5	1.23
Au-COOH(CTAB)^b^	2.9 (98)	0.13	3.2	1.25

The mean size of the nanoparticles was ^a^45 nm for Ag nanoparticles and ^b^15 nm for all types of Au nanoparticles.

**Table 2:** **Characterizations of TbADH and NADPH immobilized on Au-( *n* %)glut**^**a**^**nanoparticles**

**Glutaraldehyde ( *n* %)**	**Enzyme loading** **(mg/mL) (%)**	**Activity expressed (U/mL)**	**Specific activity (U/mg)**	**C**_**NADPH**_**(mM)**
Free TbADH	-	0.5	12.5	-
Free NADPH	-	-	-	1.6
0.3	1.8 (88)	0.23	5.8	1.2
0.5	1.8 (91)	0.27	6.2	1.23
0.7	1.8 (92)	0.36	9.0	1.26
1.0	1.9 (96)	0.35	8.7	1.25
1.4	1.8 (92)	0.13	2.6	1.2
1.8	1.8 (92)	0.1	2.6	1.2

^a^The mean size of Au nanoparticles was 15 nm, and *n* is the percent of glutaraldehyde solution used in the treatment.

**Figure 2:**
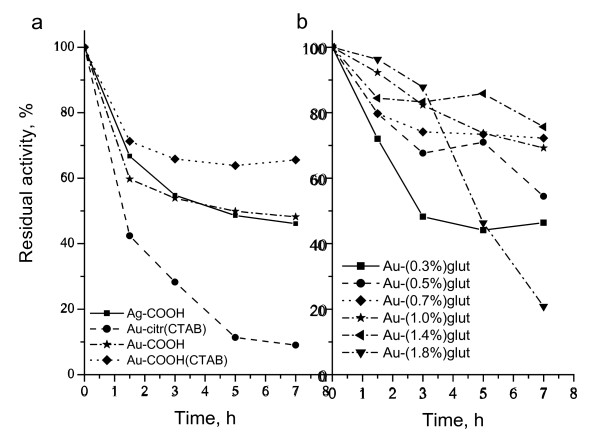
**Residual activity of TbADH during immobilization course to functionalized Ag and Au nanoparticles.** (**a**) Immobilized through physical and covalent adsorption TbADH; (**b**) immobilized through glutaraldehyde TbADH.

It is known that the amount of bound protein and its activity depend on the structure and physico-chemical properties of the carrier [10,39]. Results show that physical and covalent immobilizations through direct adsorption (Figure 1, reactions 1, 2, and 3) led to more enzyme bound to the nanoparticles surface (Table 1, Figure 2a), and less to glutaraldehyde-activated Au-NH_2_(CTAB) nanoparticles (Table 2, Figure 2b). Enzyme immobilization through physical and direct covalent adsorptions was 10% greater (97% to 98%) than immobilization through GA (88% to 96%). This is a higher amount of immobilized enzyme than reported for aminopeptidase immobilized on 64% onto Au-COOH nanoparticles [21], while similar result was obtained for glutaryl acylase immobilized on glutaraldehyde-activated sepabead supports [6].

However, immobilization promoted a decrease in enzyme activity for all experiments. The possible reason for this may be denaturation of the enzyme on nanoparticles' surface [40]. After immobilization on Au-citr(CTAB) surface, the enzyme activity decreased with about 90%, revealing that this direct immobilization method has undesirable effect on TbADH activity. The adsorbed enzyme may leak from the nanoparticles' surface during use due to the weak binding force between the enzyme and the carrier that could be caused by the transformation of amino acid residues located in the microenvironment of the catalytic site of TbADH, affecting both the local charge and topology of this site [21]. Therefore, this was a lower activity than that of glucose oxidase immobilized on Ag-citr nanoparticles [12]. The covalent immobilization through direct adsorption of the enzyme on Au-COOH, Au-COOH(CTAB), and Ag-COOH nanoparticles showed an enhancing effect on enzyme activity, loss only of 28% and 46%, respectively, of the initial enzyme activity. In that case, the binding between enzyme and nanoparticles is stronger that no leakage of the enzyme occurs. Higher activity could result from prevention of inactivation reactions with amino acid residues of the active sites.

To increase immobilization efficiency, enzyme immobilization through cross-linker glutaraldehyde was studied (Table 2, Figure 2b). In that case, the cysteamine monolayer was adsorbed on Au-citr(CTAB) nanoparticles' surface and exposed the amine group toward the solution (Figure 1, reaction 4). Then, Au-NH_2_(CTAB) nanoparticles were activated with GA (in concentration range of 0.3% to 1.8% (*v*/*v*)) through Schiff-base reaction. The TbADH molecules were bound to Au-NH_2_(CTAB) utilizing Schiff-base reaction. The key to this process is the formation of Schiff-base between aldehyde group of GA and amine group of Au-NH_2_(CTAB) nanoparticles and TbADH molecules. Increasing GA concentration in the solution of Au-NH_2_(CTAB) nanoparticles, a decreasing in enzyme activity was observed in all cases at almost constant enzyme loading (92% to 96%) in 7 h. Results showed that TbADH retained about 70% of its native activity on the treated with 0.7% and 1.0% glutraldehyde Au-NH_2_(CTAB) nanoparticles, respectively, with the highest enzyme binding (96% on Au-(1.0%)glut). A loss in enzyme activity was observed up to 1.4% GA in the solution of Au-NH_2_(CTAB) nanoparticles (Figure 2b). Although, no precipitation occurs when glutaraldehyde concentration in the solution increases. Immobilizations using GA showed a higher activity than through covalent and physical adsorptions. The presence of glutaraldehyde may be useful in the creation of additional covalent bonds between the enzyme and the nanoparticles. This usually result in a stabilization of the enzyme (Figure 2b) by preventing the enzyme leaching [6,10]. Also, higher GA concentration (up to 1.0% (*v*/*v*)) promoted loss in TbADH activity (to 80% of native activity). Similar observations have been found by others suggesting the formation of more rigid preparations with retaining of activity to some extent [6,8].

If compared, Ag-COOH, Au-COOH(CTAB), Au-(0.7%)glut, and Au-(1.0%)glut bioconjugates showed the highest activity than other bioconjugates. Also, activity of the enzyme immobilized via a 6-carbon spacer was higher than those without a spacer. Results in Tables 1 and 2 showed that immobilization through covalent adsorption led to more enzyme bound to Ag and Au nanoparticles, but is less active, than in the case of spacer-linked enzyme. Hence, for the next steps of the work that were used, these four immobilizes.

#### ** *Cofactor (NADPH) immobilization* **

Also, we have studied the possibility that functionalized Ag and Au nanoparticles could be explored as supports for cofactor NADPH immobilization. Cofactor was separately immobilized from the enzyme on Ag and Au nanoparticles through physical and covalent adsorption, which was ensured by the reactive amino group of the cofactor. Results in Tables 1 and 2 showed that about 80% of NADPH was immobilized on different types of nanoparticles that suggest a high affinity of the nanoparticles for the cofactor. This is a higher amount immobilized cofactor than reported in the literature [33,34].

### **Structural characterization of TbADH**

Although, reduced activity in the enzyme may reflect changes in conformation of the key amino acid residues around the active site of the enzyme upon immobilization [41]. Protein properties closely depend on retention of their biological activity (3D structure) [42]. Nevertheless, the retention of the protein structure and activity (protein stability) is one of the most important hurdles in successful application of different supports in biotransformation processes. Thus, we conducted investigations into the structure of TbADH immobilized on Ag and Au nanoparticles using CD and fluorescence spectroscopies.

**Figure 3:**
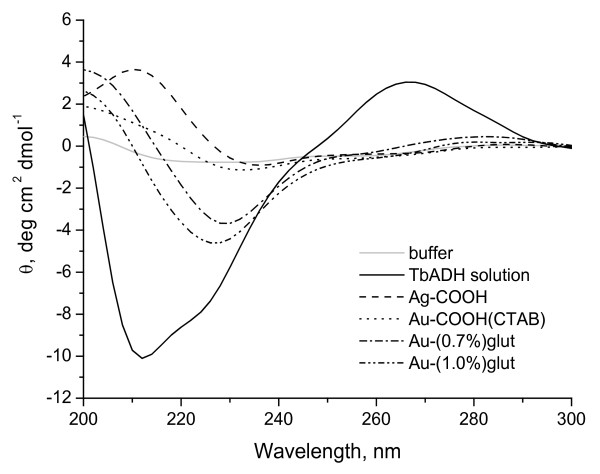
**CD spectra of the free and immobilized TbADH.** Experimental conditions: free TbADH, 140 μg/mL; immobilized TbADH, 1 mg/mL; 10-mM phosphate buffer, pH 7.0; and 25°C. Measurements were made soon after completing the immobilizations.

Secondary structure in the enzyme under the influence of Ag and Au nanoparticles is shown in Figure 3. The figure reveals the CD spectra of TbADH before and after its interaction with the nanoparticles.

TbADH is α/β-enzyme with separate α-helix and β-sheet rich regions [28]. CD spectra of such an enzyme are dominated by α-helical structure, exhibiting negative bands at around 208 and 222 nm [42]. In our experiments, CD spectrum of the native enzyme showed a dominant α-helical structure with minimums at about 210 and 222 nm. Analyses by Chen formula [43] gave results for the different types of secondary structure presented in the enzyme. We calculated 35% α-helix with less β-structure (6%) and unordered structure (2%).

CD spectra of Ag and Au nanoparticles-TbADH bioconjugates were found to be very different from the native enzyme in phosphate buffer (Figure 3). α-Helix content of the free TbADH undergoes significant change, characterized in all cases by partial loss and shift in the ellipticity at 222 nm that imply for protein unfolding [11]. CD band shifted with 8 nm (to 230 nm) for immobilized on Au-COOH(CTAB) nanoparticles enzyme and with about 13 nm (to 235 nm) for that on Ag-COOH nanoparticles. About 4- to 7-nm (to 226 and to 229 nm) shift in ellipticity at 222 nm was observed for TbADH immobilized on Au-(0.7%)glut and Au-(1.0%)glut, respectively. No precipitation occurs when TbADH interacts with the nanoparticles. The changes in the intensity and shift of the CD band could be determined by the interaction between TbADH and Ag and Au nanoparticles [12].

Clearly, the α-helix content is lower for the enzyme adsorbed on nanoparticles when compared to the free TbADH in similar solution. α-Helix content strongly decreases as the size of nanoparticles increases. These changes are significant. A large surface area of a contact will strengthen the protein-nanoparticle interaction as the contact area between the protein and nanoparticles is greater than that for the smaller nanoparticles [10]. This effect led to stronger interaction between TbADH and the larger Ag-COOH nanoparticles and result in a greater perturbation of the enzyme on 45-nm Ag NPs than on 15-nm Au NPs. Similar results have been reported for trypsin, which retains about 5% of its native α-helix content when adsorbed on silica and polystyrene [44], or for lysozyme adsorbed to the silica nanoparticles of different sizes [22].

The loss of α-helix correlated well with the observed loss of activity (Figure 2). About 28% of its activity was retained on 15-nm Au nanoparticles, while about 46% of the native activity was retained when immobilized on 45-nm Ag nanoparticles. These results suggest that the enzyme partly retains its native structure around catalytic sites [11], accompanied with retention of initial activity (about 70% for glutaraldehyde treated Au-NH_2_(CTAB) nanoparticles).

All this results imply that Ag and Au nanoparticles bind with amino acid residues of the main polypeptide chain of the enzyme which led to a change in the microenvironment of amino acid residues. However, the decreasing in α-helix was apparent with the binding of TbADH on Ag and Au nanoparticles, indicating that secondary structure of the protein was altered significantly. We observed the state (unfolded state) where α-helix of the immobilized enzyme is lower than that of the free one. In such a state, the enzyme is partly or completely denatured [22]. To complement CD experiments, we provided fluorescence measurements to investigate the effect of Ag and Au nanoparticles on tertiary structure of TbADH.

**Figure 4:**
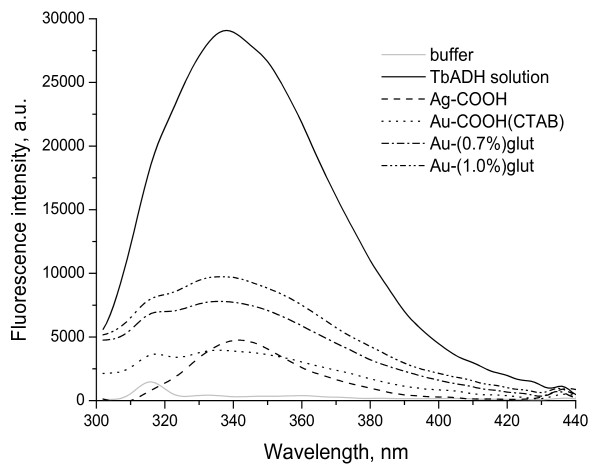
**Emission spectra of the free and immobilized TbADH.** Experimental conditions: free TbADH, 140 μg/mL; immobilized TbADH, 1 mg/mL; 10-mM phosphate buffer, pH 7; and 25°C. Measurements were made soon after completing the immobilizations.

The enzyme displays a fluorescence emission spectrum with a maximum at 338 nm (Figure 4) under our experimental conditions. This fluorescence emission maximum is characteristic of tryptophan (Trp) placed in relatively hydrophobic environment [45]. Olofsson [46] have found that TbADH possesses four tryptophan residues, partially exposed on the surface Trp14 and Trp110 and buried in the subunit Trp90 and Trp281. The authors have found that Trp14 and Trp110 remain unaffected on the changes of the surrounding environment, so any changes in fluorescence would arise from Trp90 and Trp281 residues.

Fluorescence spectra in Ag and Au nanoparticle-attached TbADH were found very different than in the native enzyme (Figure 4). Fluorescence intensity in Ag and Au nanoparticle-TbADH bioconjugates was lower than native enzyme that could be due to the interaction of the enzyme with the nanoparticles. This suggests that Ag and Au nanoparticles destabilize the native state of the enzyme to the unfolded state with decreasing the conformational stability of the enzyme and altering the microenvironment around the tryptophan residue [47]. Fluorescence intensity decreased without shifting in maximum emission wavelength (*λ*_max_) when enzyme was immobilized on Au-COOH(CTAB) and activated with 0.7% and 1.0% glutaraldehyde Au-NH_2_(CTAB) nanoparticles. Fluorescence intensity in Ag-COOH-TbADH bioconjugate was lower than the native enzyme with a significant redshift in *λ*_max_, from 338 to 342 nm. These results indicate that polarity near Trp residues is enhanced or hydrophobicity is weakened as a result of binding of Ag-COOH to TbADH [48]. Although the spectra of adsorbed TbADH have low signal-to-noise ratio, it is clear that changes in the tertiary structure of TbADH have occur upon adsorption on four types of nanoparticles. Observed changes in fluorescence intensity and *λ*_max_ can be attributed to the change in Trp environment, which alter enzyme conformation. Such an alteration was associated with a new conformational state (unfolded state) [45].

### **Application of immobilized TbADH**

The effect of immobilization on catalytic activity of TbADH was also studied to demonstrate the practicability for biocatalysing the reduction of 7-hydroxy-2-tetralone to (*S*)-7-hydroxy-2-tetralol. The results for the immobilized enzyme were compared to that for the native enzyme, which are present in Table 3.

**Table 3:** **Summary of conversions and *ee* of ( *S* )-7-hydroxy-2-tetralol synthesized with different immobilization systems**

**Immobilization system**	**Yield**^**a**^**(%)**	** *ee* **^**b**^**, %**
Buffer	>99	>99
Ag-COOH	93	>99
Au-COOH(CTAB)	93	>99
Au-(0.7%)glut	>99	>99
Au-(1.0%)glut	>99	>99

^a^Determined after 48 h, and ^b^in all cases (*S*)-enantiomer was synthesized.

Results showed that the activity of enzyme was not enhancing on immobilization process. Cofactor NADPH was also immobilized separately from the enzyme on each type of nanoparticles in reduced form to enable reduction of 7-hydroxy-2-tetralone. Excellent results were obtained leading to the total consumption of the substrate (>93% conversion) (Table 3). Enantiomeric purity of the formed (*S*)-7-hydroxy-2-tetralol in all cases was >99%. When the cofactor was regenerated, the enzyme worked continuously for about 2 days. de Temino [2], have demonstrated that alcohol dehydrogenase from *Lactobacillus kefir* entrapped in PVA-stabilized two-phase system can be successfully employed for producing chiral alcohols with high enantiomeric excess (>90%) but with lower conversion (between 40% and 100%). They suggest that activity of the enzyme should increase proportionally to the decrease of the bead diameter due to the favorable diffusion. In our experiments, only non-significant difference of 3% to 4% in conversions for different systems was observed. Ag and Au nanoparticles showed high affinity for NADPH, about 80% immobilization. Catalytic activity was observed in all cases that suggest that enzyme activity was not affected by enzyme bonding to the immobilized cofactor. Although the immobilized TbADH activity decreased significantly, the immobilized TbADH had a good productivity. This reveals that four-type nanoparticles indeed could be practically used for the immobilized enzymes.

### **Storage stability of immobilized TbADH**

**Figure 5:**
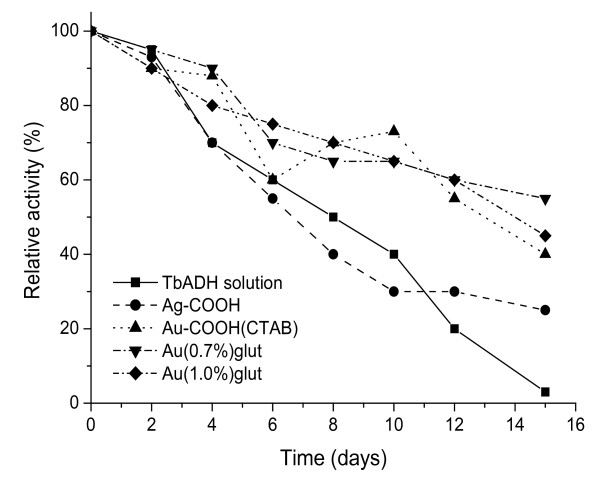
Comparison of storage stabilities of the free and immobilized TbADH.

To complete the characterization of the immobilized TbADH, the activity was measured as a function of long-term storage time at 4°C. Compared to the free enzyme, immobilized one showed lower activity (Tables 1 and 2). Storage stability in buffer and immobilized on Ag and Au nanoparticles TbADH is shown in Figure 5.

Storage stability of nanoparticle-attached enzyme determined the usefulness of such preparations. Figure 5 illustrates long-term storage stability of the free and immobilized enzyme at 4°C in 100-mM phosphate buffer, pH 7.0. Residual activity was measured every 2 days. Decreasing in the enzyme activity was observed in all cases. Compared to Ag-COOH-TbADH and Au-COOH(CTAB)-TbADH, the activity of TbADH on Au-(*n*%)glut decreased slower and remained more than 50% after 15 days at 4 °C. However, under the same conditions, a nearly complete inactivation of free TbADH was observed (loss to 97% of the initial activity). Results indicate that immobilization enhanced the stability of TbADH. For example, lipase encapsulated within silica-PEG matrix retained to 50% of its activity after storage for 94 days [49], while lactate dehydrogenase entrapped in double-walled carbon nanotube-alginate gel retained to 75% activity after 1-month storage [40].

## Conclusion

Citrate-stabilized Ag and Au nanoparticles, followed by post-modifications, were synthesized and characterized using TEM and UV–vis spectroscopy. Nanoparticles were of nanosize with a diameter of 15 and 45 nm for Au-citr and Ag-citr nanoparticles, respectively, and showed characteristic absorption bands. The nanoparticles were efficient as nanoadsorbend for TbADH (>90%) and cofactor NADPH (>80%) immobilizations. CD and fluorescence spectroscopies were suitable for characterizing the enzyme-nanoparticle interaction. Immobilization process induced different conformational state with a strong denaturation of the enzyme. A strong decreasing and shift in the ellipticity and emission maximum were observed. In these cases, activity of a given preparation depends on conformational state of the enzyme (loss to 40% to 50% of initial activity). Immobilized on Ag and Au nanoparticles, TbADH and NADPH were successfully used in the production of alcohols without loss of productivity and optical purity. This method could be used for immobilization of other proteins too and also has a potential to be useful for the development of biosensors for alcohol detection.

## Competing interests

The authors declare that they have no competing interests.

## Authors' contributions

GAP carried out the immobilization and structural studies of TbADH and drafted the manuscript. KZ and PŽ carried out the synthesis and characterization of gold and silver nanoparticles and participated in the interpretation of experimental data. VK supervised and participated in discussion of the manuscript. All authors read and approved the final manuscript.

## Supplementary Material

Additional file 1: **RP- and chiral HPLC analyses.** Description: RP- and chiral HPLC conditions for the separation of 7-hydroxy-2-tetralone and7-hydroxy-2-tetralol. Click here for file

Additional file 2: Figure 1 Title: Characterization of silver and gold nanoparticles. Description: UV–vis and TEM images of Ag-citr and Au-citr-stabilized nanoparticles [50]. Click here for file
