# Supplementary material for: Gold and silver nanoparticles for biomolecule immobilization and enzymatic catalysis
Source: Nanoscale Res Lett. 2012 Jun 1;7(1):287. doi: 10.1186/1556-276X-7-287 (PMC3447686; doi:10.1186/1556-276X-7-287)
Supplement: Additional file 1: — RP- and chiral HPLC analyses. Description: RP- and chiral HPLC conditions for the separation of 7-hydroxy-2-tetralone and7-hydroxy-2-tetralol. [file 1556-276X-7-287-S1.pdf]

Additional file 1

HPLC conditions for 7-hydroxy-2-tetralone and 7-hydroxy-2-tetralol separation.

RP-HPLC analyses were recorded with LC 5000 HPLC system (INGOS, Czech Republic). Quantification of ketone 7-hydroxy-2-tetralone to alcohol 7-hydroxy-2-tetralol conversions was completed using Wakosile C18 column (SGE). Mobile phase was 30/70 (v/v) acetonitril/water at a flow rate 0.5 mL min^{-1}.

Chiral HPLC analyses of 7-hydroxy-2-tetralol were provided on Cyclobond I™ 2000 SN column (SUPELCO, USA) using 1/99 (v/v) acetonitril/0.1% TEAA (pH 4.0) at 0.8 mL min^{-1}.

All HPLC analyses were monitored at 272 nm.
