# Supplementary material for: Gold and silver nanoparticles for biomolecule immobilization and enzymatic catalysis
Source: Nanoscale Res Lett. 2012 Jun 1;7(1):287. doi: 10.1186/1556-276X-7-287 (PMC3447686; doi:10.1186/1556-276X-7-287)
Supplement: Additional file 2: Figure 1 — Title: Characterization of silver and gold nanoparticles. Description: UV–vis and TEM images of Ag-citr and Au-citr-stabilized nanoparticles [50]. [file 1556-276X-7-287-S2.pdf]

Additional file 2

Characterization of silver and gold nanoparticles.

Monodispersed Ag-citr and Au-citr nanoparticles had spherical form with absorption maximum of 520 nm for Au-citr nanoparticles and 430 nm for Ag-citr nanoparticles, that correspond well to the estimated by transmission electron microscope (TEM) average diameters of 15 nm for Au-citr nanoparticles and 45 nm for Ag-citr nanoparticles [37,50].

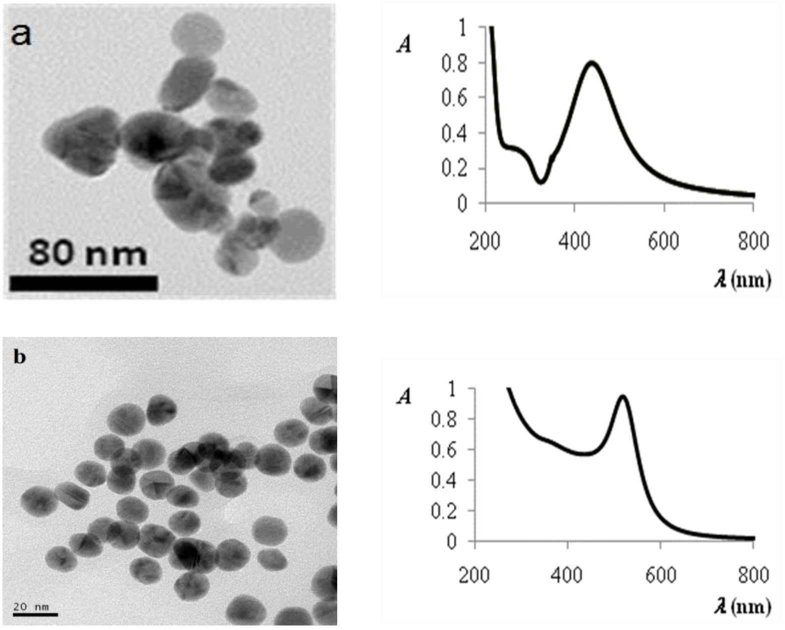

Figure S1. TEM images and UV-Vis absorption spectra obtained for citrate-reduced (a) silver and (b) gold nanoparticles.
